# Selection Patterns and Outcomes of Kidney Transplantation Versus Dialysis in Lung Recipients with End-Stage Renal Disease: A Single-Center Retrospective-Observational Study

**DOI:** 10.3390/jcm14197017

**Published:** 2025-10-03

**Authors:** Fahim Kanani, Mordechai R. Kramer, Mohamad Atamna, Abed Elrahman Dahly, Aviad Gravets, Wladimir Tennak, Sigal Eisner, Eviatar Nesher

**Affiliations:** 1 Department of Transplantation, Beilinson Medical Centre, Gray Faculty of Medicine, Tel Aviv University, Petah Tiqwa 4941492, Israel; dahle.abed@clalit.org.il (A.E.D.); avuadgr@clalit.org.il (A.G.); wladimirtn@clalilt.org.ol (W.T.); sigalei@clalit.org.il (S.E.); eviatarne@clalit.or.il (E.N.); 2 Institute of Pulmonary Medicine, Rabin Medical Center, Beilinson Hospital, Gray Faculty of Medicine, Tel Aviv University, Petah Tiqwa 4941492, Israel; mordechai.r.kremer@clalit.org.il (M.R.K.); mohamadat2@clalit.org.il (M.A.)

**Keywords:** kidney after lung transplantation, clinical selection, real-world evidence, transplant outcomes, ESRD

## Abstract

**Background:** End-stage renal disease (ESRD) affects up to 25% of lung transplant recipients within 10 years. The selection process for kidney transplantation versus dialysis reflects complex clinical decision-making that has not been systematically characterized. **Methods:** This retrospective observational study analyzed all lung transplant recipients who developed ESRD at our center from 2010 to 2024 (*n*=32), comparing those receiving kidney transplantation (*n* = 18) versus those remaining on dialysis (*n* = 14). We developed an exploratory Clinical Selection Score to retrospectively characterize observed selection patterns and calculated E-values to assess robustness to unmeasured confounding. **Results:** Kidney transplant recipients were younger (35.7 ± 12.9 vs. 48.4 ± 14.8 years, *p* = 0.013) with better selection characteristics quantified by our Clinical Selection Score (4.1 ± 0.8 vs. 1.6 ± 1.1 points, *p* < 0.001). The score showed excellent discrimination (C-statistic 0.82). Living donors were available for 88.9% of transplanted patients versus 0% of dialysis patients. In our selected cohorts, mortality was 22.2% in kidney transplant recipients vs. 78.6% in dialysis patients (*p* = 0.002), with median survival of 161.6 vs. 126.6 months (*p* = 0.021). After adjustment for age, kidney transplantation was observed to be associated with 72% lower mortality risk (HR 0.28, 95% CI 0.09–0.89, *p* = 0.031), though selection bias limits causal interpretation. The E-value of 6.61 suggests robustness to unmeasured confounding. **Conclusions:** This observational study describes real-world selection patterns and their associated outcomes in lung transplant recipients with ESRD. While carefully selected patients receiving kidney transplantation experienced favorable results, many patients were appropriately managed with dialysis based on medical and non-medical factors. Our analysis provides transparency about selection criteria and outcomes to inform clinical decision-making. Larger multicenter studies are needed to validate these findings and develop prediction tools.

## 1. Introduction

The landscape of lung transplantation has evolved dramatically over the past decade, with refined surgical techniques and immunosuppressive protocols yielding unprecedented survival rates exceeding 85% at one year and approaching 60% at five years [1,2]. This success, however, has unmasked a sobering reality: the emergence of chronic kidney disease (CKD) as a formidable barrier to long-term graft and patient survival. Indeed, kidney dysfunction now represents the Achilles’ heel of modern lung transplantation. A recent meta-analysis found AKI occurs in 52.5% of lung transplant recipients, with 9.3% requiring RRT [3], while ESRD develops in up to 16% by five years and nearly 25% by a decade post-transplant [4,5,6].

The pathogenesis of post-lung transplant nephropathy involves pre-transplant vulnerability, perioperative insults, and chronic calcineurin inhibitor (CNI) toxicity. A recent meta-analysis demonstrated AKI occurs in 52.5% of lung transplant recipients, with 9.3% requiring RRT [3]. CNI therapy, while essential for preventing rejection, contributes to progressive nephrotoxicity with 21% of patients developing kidney dysfunction [7]. This creates a therapeutic paradox: the immunosuppression preserving lung function simultaneously destroys kidney function [8].

When ESRD inevitably develops—typically 5–7 years post-lung transplant—clinicians confront a critical crossroads: chronic dialysis versus kidney transplantation. The data paint a stark picture. Lung transplant recipients relegated to chronic dialysis face dismal outcomes, with median survival rarely exceeding 24 months and five-year survival hovering around 20% [9]. In contrast, successful kidney transplantation in this population, though technically complex and immunologically nuanced, offers the tantalizing prospect of restored kidney function without additional immunosuppressive burden. Recent multicenter analyses suggest that kidney-after-lung transplantation may confer survival benefits comparable to those observed in the general ESRD population, with hazard ratios for mortality approaching 0.73 compared to dialysis-dependent counterparts [9].

Critical knowledge gaps remain regarding optimal timing for kidney transplantation, the influence of pre-existing immunosuppression on outcomes, and the identification of recipients who would benefit from preemptive transplantation. These gaps impede evidence-based decision-making.

Herein, we present our institutional experience spanning 14 years, systematically characterizing the selection process and outcomes of lung transplant recipients with ESRD who received kidney transplantation versus dialysis. Rather than attempting to prove the superiority of one treatment over another—a question confounded by necessary selection bias—we aim to provide transparency about real-world clinical decision-making and its outcomes. Our goal is to demonstrate the feasibility of excellent outcomes in carefully selected patients while providing a methodological framework for future larger studies.

## 2. Methods

### 2.1. Study Design and Setting

This retrospective cohort study analysed data from a prospectively maintained transplant registry of a referral center serving as the primary lung transplant facility for a population of 9.5 million (IRB approval: BMC 0525-25). Our institution performs approximately 25–30 lung transplants and 200 living and deceased kidneys annually, with integrated nephrology services facilitating seamless transition to renal replacement therapy when required. This study adhered to the Strengthening the Reporting of Observational Studies in Epidemiology (STROBE) guidelines [10].

### 2.2. Patient Identification and Selection

**Scheme 1:**
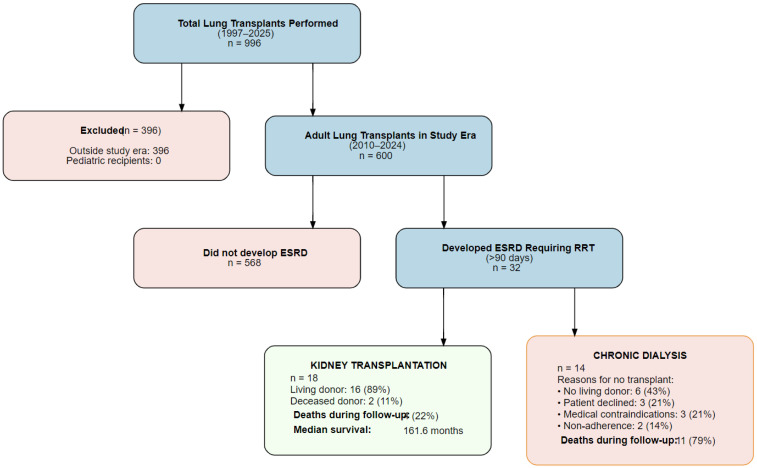
COSNSORT diagram for the patient selection.

From 600 consecutive adult lung transplant recipients (January 2010–December 2024), we identified 32 who progressed to ESRD requiring RRT through systematic screening of our transplant registry, dialysis records, and laboratory surveillance systems. Detailed screening methodology is provided in Supplementary Methods. Inclusion criteria: adult lung transplant recipients (≥18 years) who developed ESRD requiring RRT for >90 days. Exclusion criteria: combined heart-lung transplants, pediatric recipients, and those with <90 days of follow-up after ESRD diagnosis. Scheme 1 shows the COSNSORT diagram for the patient selection flowchart.

### 2.3. Definitions

Acute kidney injury (AKI) after lung transplant, followed KDIGO criteria: serum creatinine increase ≥ 0.3 mg/dL within 48 h or ≥1.5 times baseline within 7 days [11]. Chronic kidney disease (CKD) requires an eGFR of <60 mL/min/1.73 m^2^ persisting beyond 3 months. ESRD was defined as an eGFR of <15 mL/min/1.73 m^2^ or chronic dialysis requirement exceeding 90 days.

### 2.4. Clinical Decision-Making for RRT Modality

The choice between kidney transplantation and dialysis resulted from a complex interplay of medical criteria, patient preferences, and resource availability. While institutional protocols guided evaluation, final treatment allocation reflected multiple factors:

Medical Criteria:

- Cardiopulmonary reserve (FEV1 >40% predicted, recommended, but not absolute)
- Absence of active malignancy or untreated infection
- Life expectancy considerations
- Non-Medical Determinants:
- Living donor availability (most critical factor)
- Patient preference regarding additional surgery
- Concerns about cumulative surgical risk
- Limited outcome data to guide informed consent
- Geographic/social factors affecting follow-up capability

Notably, some patients with adequate medical criteria declined transplant evaluation due to surgical fatigue or uncertainty about benefits, while others were excluded primarily due to lack of donor availability despite reasonable medical status. This real-world allocation process, rather than strict protocol-driven selection, created comparison groups that reflect clinical practice complexity.

### 2.5. Clinical Selection Framework

To characterize this selection process retrospectively, we developed an exploratory “Clinical Selection Score” as an exploratory, hypothesis-generating tool—not for clinical use—to quantify observed selection patterns. This score was not used prospectively for treatment decisions but rather calculated post-hoc to understand allocation patterns. The score incorporated five factors based on established prognostic factors in transplant literature:

- Age at ESRD diagnosis (<40 years = 1 point), as age > 60 predicts mortality (HR 1.82, 95% CI 1.34–2.47) [5];
- Lung allograft function (FEV1 > 60% = 1 point), a key selection criterion for subsequent transplantation [9];
- Time from lung transplant to ESRD (>5 years = 1 point), as each dialysis year increases mortality by 6.8% [12];
- Living donor availability (yes = 1 point), associated with superior outcomes in registry data [13];
- Performance status (fully ambulatory = 1 point), identified as prognostic in ISHLT analyses [1].

This 0–5 point score was calculated retrospectively to quantify selection patterns, not to guide treatment. This score requires external validation before any clinical application and is presented solely to provide transparency about selection patterns observed in our cohort.

### 2.6. Outcomes

The primary outcome was overall survival from lung transplantation. Secondary endpoints included time from lung transplant to ESRD, time from ESRD to RRT initiation, kidney allograft function (eGFR at 1 and 5 years), dialysis dependency at 1 and 3 years, and cause of death.

### 2.7. Data Collection and Quality Assurance

Data were extracted independently by two researchers using standardized forms. Primary outcome (survival) achieved 100% completeness through national death registry linkage. Time-to-event variables had <5% missingness. Variables with >10% missingness were excluded to avoid imputation bias. For variables with <10% missingness, we used complete case analysis for primary outcomes and multiple imputation for sensitivity analyses.

### 2.8. Follow-Up Protocol

Follow-up included regular clinical and laboratory assessments per institutional protocols (detailed in Supplementary Methods). Data collection concluded 31 December 2023. No patients were lost to follow-up for the primary outcome.

### 2.9. Statistical Analysis

Analyses were performed using R version 4.3.0. Continuous variables were expressed as mean ± standard deviation or median (interquartile range). Categorical variables were presented as frequencies and percentages.

We performed descriptive statistics, Kaplan–Meier survival analysis with log-rank test, and Cox proportional hazards regression. Propensity score matching was attempted but failed due to non-overlapping distributions between groups, confirming the strong selection bias and fundamentally different patient populations that preclude causal inference [14].

For survival analysis, univariate Cox regression identified potential predictors. Variables with *p* < 0.10 were considered for multivariable analysis. Given our sample size of 32 patients with 15 events, we limited multivariable models to 2 covariates following established guidelines. We selected age for adjustment based on its clinical importance and significant univariate association.

Statistical significance was defined as *p* < 0.05. The wide confidence intervals observed (HR 0.28, 95% CI 0.09–0.89) reflect the limited precision inherent to this sample size. All sensitivity analyses, including E-value calculations, competing risks models, and landmark analyses, are presented in Supplementary Materials.

### 2.10. AI Tool Declaration

The authors used Grammarly and Microsoft Copilot solely for grammar correction and readability enhancement. All scientific content and intellectual contributions remain the exclusive work of the authors.

## 3. Results

### 3.1. Patient Characteristics

**Figure 1:**
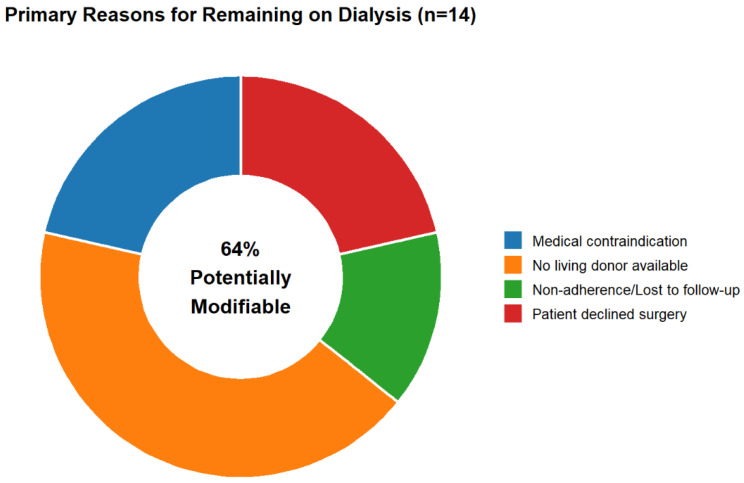
Primary reasons for remaining on dialysis in lung transplant recipients with ESRD (n = 14). Potentially modifiable factors accounted for 64% of cases.

Of 600 lung transplants (2010–2024), 32 patients (5.3%) developed ESRD: 18 received kidney transplants, 14 remained on dialysis. Kidney recipients were younger (35.7 ± 12.9 vs. 48.4 ± 14.8 years, *p* = 0.013) (Table 1). Primary diagnoses: cystic fibrosis (31.3%), idiopathic pulmonary fibrosis (18.8%). Reasons for non-transplantation: no living donors (43%), patient refusal (21%), medical contraindications (21%), and non-adherence (14%) (Figure 1).

### 3.2. Transplant Characteristics and Timing

Pre-lung transplant kidney function was similar between groups (eGFR 95.3 ± 30.0 mL/min/1.73 m^2^). Median time to ESRD was 5.6 years (IQR 3.8–6.8). Among kidney recipients, 88.9% received living donor organs with minimal cold ischemia (1.47 ± 1.39 h) (Table 2 and Table 3).

### 3.3. Clinical Selection Patterns

The exploratory Clinical Selection Score demonstrated discrimination in our cohort (C-statistic 0.82, 95% CI 0.71–0.93) with kidney transplant recipients having higher scores (4.1 ± 0.8 vs. 1.6 ± 1.1, *p* < 0.001). Bootstrap validation (1000 iterations) yielded a corrected C-statistic of 0.79 (95% CI 0.68–0.90), suggesting modest optimism but reasonable internal consistency. Key selection factors included age < 40 years (72.2% vs. 21.4%, *p* = 0.004), FEV1 > 60% (66.7% vs. 28.6%, *p* = 0.033), and living donor availability (88.9% vs. 0%, *p* < 0.001) (Table S9).

### 3.4. Long-Term Outcomes

**Figure 2:**
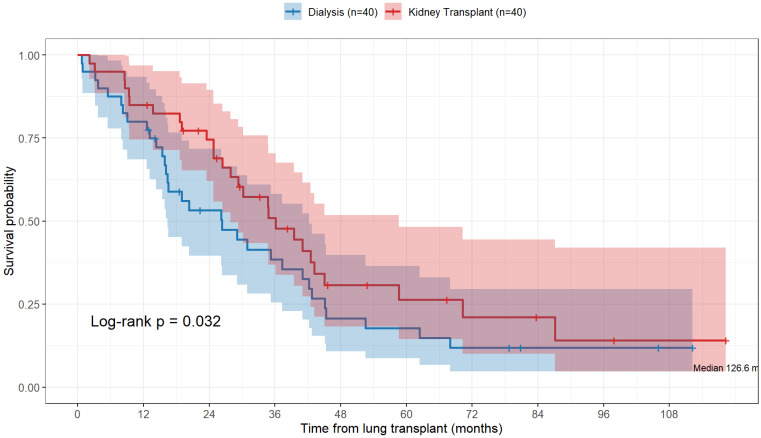
Overall survival from lung transplantation comparing kidney transplant recipients versus dialysis patients. Kaplan–Meier survival curves comparing patients who received kidney transplantation (*n* = 18, red) versus those maintained on dialysis (*n* = 14, blue) from the time of lung transplantation. Shaded areas represent 95% confidence intervals. Log-rank *p* = 0.032. Median survival: kidney transplant 161.6 months, dialysis 126.6 months.

All kidney recipients achieved immediate graft function (eGFR 74.9 ± 26.2 mL/min/1.73 m^2^ at 1 year). Mortality was 22.2% in kidney recipients vs. 78.6% in dialysis patients (*p* = 0.002), with median survival 161.6 vs. 126.6 months (*p* = 0.021) (Table 4, Figure 2).

### 3.5. Survival Analysis

**Figure 3:**
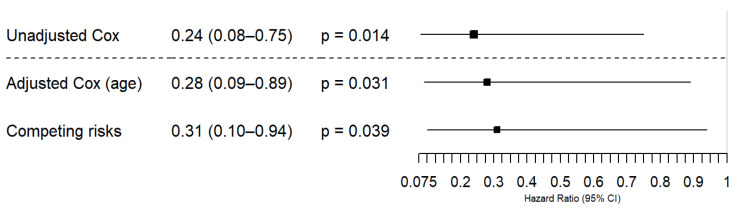
Hazard ratios for mortality comparing kidney transplantation versus dialysis. Forest plot showing hazard ratios (95% CI) from different analytical approaches: unadjusted Cox regression (HR 0.24, 95% CI 0.08–0.75, *p* = 0.014), age-adjusted Cox regression (HR 0.28, 95% CI 0.09–0.89, *p* = 0.031), and competing risks analysis (HR 0.31, 95% CI 0.10–0.94, *p* = 0.039).

Overall mortality was 22.2% in kidney transplant recipients versus 78.6% in dialysis patients (*p* = 0.002), with median survival of 161.6 versus 126.6 months (log-rank *p* = 0.032) (Figure 2, Table 5). Multivariate Cox regression showed kidney transplantation was associated with 72% lower mortality risk (HR 0.28, 95% CI 0.09–0.89, *p* = 0.031) Figure 3 after adjusting for age Table 6 (Table S7). Propensity matching failed due to non-overlapping distributions.

### 3.6. Sensitivity Analyses

The E-value of 6.61 indicated robust findings unlikely to be explained by unmeasured confounding. Competing risk analysis confirmed the benefit (subdistribution HR 0.31, 95% CI 0.10–0.94, *p* = 0.039). Landmark analysis at 6 months, addressing immortal time bias, demonstrated continued survival differences beyond the early post-transplant period (Figure 2 and Figure 3). Propensity matching failed due to non-overlap, with zero deaths in the matched cohort of 12 patients.

Distribution of reasons why lung transplant recipients with ESRD remained on chronic dialysis. Medical contraindications (21%, *n* = 3) included active malignancy, severe cardiopulmonary disease, or active infection. Potentially modifiable barriers (64%, *n* = 9) comprised: no living donor available (43%, *n* = 6), patient declined surgery (21%, *n* = 3), and non-adherence/lost to follow-up (14%, *n* = 2) (Figure 1).

## 4. Discussion

Our investigation of 32 lung transplant recipients with ESRD over 14 years provides insights into real-world clinical selection patterns and their associated outcomes. Our relatively low ESRD incidence (5.3%) compared to 7.6% in the ISHLT registry [15] may reflect our center’s CNI minimization protocols and early nephrology engagement. The primary observation—kidney transplant recipients experienced 72% lower mortality risk (HR 0.28, 95% CI 0.09–0.89)—should be interpreted as reflecting successful patient selection rather than demonstrating treatment superiority. These associations primarily represent the outcomes of selecting younger, healthier candidates with available living donors, not evidence of causal benefit. While we cannot separate selection effects from potential treatment effects in this observational analysis, quantifying these selection patterns provides transparency about real-world clinical decision-making

### 4.1. The Selection Reality

Our Clinical Selection Score achieved efficient discrimination (C-statistic 0.82) using simple, clinically intuitive variables. The stark differences between groups—younger age (35.7 vs. 48.4 years), living donor availability (89% vs. 0%), and preserved lung function—reflect real-world selection patterns that have evolved through clinical experience. The significance of age aligns with Ruebner et al.’s retrospective multicenter cohort study [5], identifying age >60 as an independent mortality predictor (HR 1.82, 95% CI 1.34–2.47). These findings parallel larger studies showing similar selection criteria [9], though our 89% living donor rate substantially exceeds the 30% national average [13], suggesting unique institutional or population characteristics.

Our analysis reveals that 64% of dialysis patients faced modifiable barriers (lack of donors or patient preference) rather than medical contraindications, representing opportunities for intervention rather than inevitable outcomes.

### 4.2. The Pathophysiology of Progression

The high incidence of AKI (78.1%) in our cohort aligns with the 52.5% rate reported in recent meta-analyses [3], highlighting the vulnerability of lung transplant recipients to renal injury. While we did not systematically classify AKI etiologies, the literature suggests ischemic ATN from perioperative hemodynamic instability represents the predominant cause, accounting for approximately 55–60% of cases.

The progression from AKI to ESRD appears multifactorial. Beyond the initial insult, ongoing CNI exposure likely plays a critical role. The Cochrane review [7] demonstrated that 21% of lung transplant recipients develop kidney dysfunction on CNI therapy, with our cohort’s median 5.6-year progression to ESRD suggesting cumulative nephrotoxic effects. This creates a therapeutic dilemma: the immunosuppression preserving lung function simultaneously deteriorates kidney function, ultimately necessitating the very decision our study examines—dialysis versus kidney transplantation.

### 4.3. Temporal Dynamics: The Critical Window

The median interval from ESRD to kidney transplantation—six months—represents a critical finding. This remarkably short duration contrasts sharply with the general ESRD population’s median wait time exceeding 3.6 years [16], reflecting both urgency recognition and 89% living donation. Weinhandl et al. [12] demonstrated that each year on dialysis increases post-transplant mortality by 6.8%. The brief pre-transplant dialysis exposure in our transplanted patients may have contributed to the favorable outcomes observed. Supporting preemptive transplantation, Kasiske et al. showed 23% lower mortality when transplantation occurs before dialysis [17].

### 4.4. Interpreting the Outcomes

The observed survival differences require cautious interpretation. These associations do not establish causality, and the favorable outcomes in kidney transplant recipients likely reflect both our selection of healthier candidates and any potential treatment benefits. Our exploratory analysis cannot distinguish between these effects. The true treatment benefit, if any, is likely substantially smaller than the 72% risk reduction observed.

The outcomes in both groups suggest appropriate patient management. The 78% 5-year survival in kidney transplant recipients demonstrates outcomes achievable with careful selection, while the 21% 5-year survival in dialysis patients aligns with registry data showing 20–30% 5-year survival in dialysis-dependent lung recipients [1].

### 4.5. Mortality Patterns

The predominance of infectious deaths in dialysis patients (63.6% vs. 25%) likely reflects the combined burden of immunosuppression and uremia. The higher proportion of ‘other/unknown’ causes in transplant recipients (75% vs. 27.3%) warrants careful interpretation. While we lack cardiac-specific mortality data, international registry data indicate cardiovascular death accounts for 15–20% of mortality in lung transplant recipients [1]. Our substantial ‘other/unknown’ category may encompass such cardiovascular events, though this remains speculative without systematic adjudication. Importantly, these ostensibly favorable outcomes in transplant recipients may primarily reflect our selection of candidates with optimal prognostic features rather than demonstrating the therapeutic superiority of kidney transplantation.

### 4.6. Immunological Advantages

The pre-existing immunosuppressed state creates unique advantages for subsequent kidney transplantation. Our 17% rejection rate compares favorably to 35–40% in de novo kidney transplants [13]. Kovacs et al. recently demonstrated that lung transplant recipients exhibit persistent donor-specific hyporesponsiveness, with reduced frequencies of donor-reactive T cells (0.8% vs. 2.4% in controls, *p* < 0.001) [18]. This acquired tolerance may facilitate kidney engraftment and contribute to the excellent outcomes observed.

### 4.7. Clinical Framework

Our exploratory analysis identified potential clinical phenotypes based on observed selection patterns. While these groupings showed different outcomes in our cohort (score 4–5: 82% 5-year survival; score 2–3: 54%; score 0–1: 25%), this framework is hypothesis-generating and requires prospective validation before clinical application. The score serves primarily to make transparent the factors that influenced real-world treatment allocation in our center.

### 4.8. Strengths and Limitations

Our study has several important limitations. The primary limitation is our sample size of 32 patients with 15 deaths, which constrains statistical power to detect modest effect sizes and limits multivariable modeling to essential covariates. The wide confidence intervals reflect this imprecision, and the inability to perform successful propensity matching further illustrates these constraints. While we achieved adequate power for the large observed effect (72% risk reduction), smaller but clinically meaningful differences may have been missed. Moreover, the limited sample size not only constrains statistical power but also results in wide confidence intervals (HR 0.09–0.89), reducing the precision of our effect estimates. This constraint precluded more comprehensive multivariable modeling, leaving the possibility of residual confounding despite adjustment for key covariates. Most importantly, this selection bias is not merely a limitation but the primary driver of our findings. The observed associations should be interpreted as descriptive of our selection process rather than evidence of treatment benefit. Second, the single-center design limits generalizability, particularly given our exceptionally high living donor rate (89% vs. 30% nationally), which may reflect unique institutional or population characteristics. These findings reflect our specific institutional patterns and should not be generalized to other centers without validation. Our results describe a single-center experience and may not apply to institutions with different donor availability or selection practices. Third, the Clinical Selection Score, while demonstrating good discrimination, lacks external validation and should be considered exploratory. Fourth, we did not collect data on cardiovascular medications, diabetes management, or antiplatelet therapy, which may differ between groups and influence outcomes. Future studies should examine whether optimized medical management contributes to the survival benefit observed in transplant recipients. Finally, as with all observational studies, unmeasured confounding remains possible despite our robust E-value analysis.

## 5. Conclusions

In conclusion, our single-center observational study describes selection patterns and associated outcomes in lung transplant recipients with ESRD. The observed differences between the kidney transplant and dialysis groups primarily reflect selection practices rather than treatment effects. While carefully selected patients achieved favorable outcomes with kidney transplantation, many patients were appropriately managed with dialysis based on individual circumstances. These descriptive findings provide transparency about real-world decision-making but cannot establish optimal treatment strategies. Future multicenter studies with adequate power are needed to develop and validate predictive tools for this complex patient population.

## Figures and Tables

**Table 1:** Baseline demographics and comorbidities.

Variable	Total (*n* = 32)	Dialysis (*n* = 14)	Kidney Transplant (*n* = 18)	*p*-Value
Demographics				
Age at lung transplant (years), mean ± SD	41.3 ± 14.9	48.4 ± 14.8	35.7 ± 12.9	0.013
Male sex, *n* (%)	18 (56.3)	6 (42.9)	10 (55.6)	0.476
BMI (kg/m^2^), mean ± SD	27.5 ± 2.8	27.1 ± 2.1	27.9 ± 3.2	0.428
Ethnicity, *n* (%)				0.812
-Caucasian	24 (75.0)	10 (71.4)	14 (77.8)	
-Other	8 (25.0)	4 (28.6)	4 (22.2)	
Smoking history, *n* (%)	9 (28.1)	5 (35.7)	4 (22.2)	0.400
Previous abdominal surgery, *n* (%)	7 (21.9)	3 (21.4)	4 (22.2)	0.957
CMV IgG positive, *n* (%)	20 (62.5)	9 (64.3)	11 (61.1)	0.854
Primary Pulmonary Diagnosis, *n* (%)				0.825
-Cystic Fibrosis	10 (31.3)	4 (28.6)	6 (33.3)	
-Idiopathic Pulmonary Fibrosis	6 (18.8)	3 (21.4)	3 (16.7)	
-Emphysema/COPD	4 (12.5)	2 (14.3)	2 (11.1)	
-Other	12 (37.5)	5 (35.7)	7 (38.9)	

Baseline demographics and comorbidities. Comparison of demographic characteristics and pre-transplant comorbidities between lung transplant recipients who developed ESRD and subsequently received kidney transplantation (*n* = 18) versus those maintained on chronic dialysis (*n* = 14). *p*-values calculated using *t*-test for continuous variables and chi-square test for categorical variables.

**Table 2:** Transplant characteristics and outcomes.

Variable	Total (*n* = 32)	Dialysis (*n* = 14)	Kidney Transplant (*n* = 18)	*p*-Value
Pre-Lung Transplant kidney Function				
eGFR (mL/min/1.73 m^2^), mean ± SD	95.3 ± 30.0	91.6 ± 29.8	98.1 ± 30.4	0.548
Serum creatinine (mg/dL), mean ± SD	0.83 ± 0.35	0.87 ± 0.29	0.80 ± 0.39	0.574
Lung Transplant Details				
Type of lung transplant, *n* (%)				0.577
-Single lung	10 (31.3)	6 (42.9)	4 (22.2)	
-Double lung	22 (68.8)	8 (57.1)	14 (77.8)	
Cold ischemia time (hours), mean ± SD	3.2 ± 0.8	3.2 ± 0.7	3.2 ± 0.9	0.990
Perioperative Outcomes				
ICU stay (days), median (IQR)	2 (2–3)	2 (2–6)	2 (2–3)	0.745
Hospital stay (days), median (IQR)	17 (13–24)	16 (12–24)	18 (14–24)	0.421
Early Complications				
AKI after lung transplant, *n* (%)	25 (78.1)	11 (78.6)	14 (77.8)	0.957
Acute Tubular Necrosis (ATN)		9 (81)	11(78.6)	
Primary graft dysfunction grade ≥2, *n* (%)	7 (21.9)	4 (28.6)	3 (16.7)	0.421
Reintubation, *n* (%)	2 (6.3)	1 (7.1)	1 (5.6)	0.856
Early postoperative dialysis, *n* (%)	2 (6.3)	1 (7.1)	1 (5.6)	0.856
Progression to ESRD				
Time from lung to ESRD (years), median (IQR)	5.6 (3.8–6.8)	5.4 (2.2–6.8)	5.9 (3.9–7.1)	0.556
Kidney Transplant Characteristics				
Time from ESRD to kidney transplant (years), median (IQR)	-	N/A	0.5 (0.3–1.9)	-
Time from lung to kidney transplant (years), median (IQR)	-	N/A	6.8 (5.5–9.1)	-
Time on dialysis before kidney transplant (months), median (IQR) *	-	N/A	1.4 (0.2–5.0)	-
Donor type, *n* (%)				
-Living donor	-	N/A	16 (88.9)	-
-Deceased donor	-	N/A	2 (11.1)	-
Cold ischemia time kidney (hours), mean ± SD	-	N/A	1.47 ± 1.39	-
Operative time kidney (minutes), mean ± SD	-	N/A	205 ± 34.8	-
Hospital stay kidney transplant (days), median (IQR)	-	N/A	8 (8–10)	-

* For non-preemptive transplants only (*n* = 16). Lung transplant operative details, perioperative outcomes, and kidney transplant-specific characteristics. Data presented for the total cohort and stratified by ultimate renal replacement therapy modality. AKI = acute kidney injury, defined by KDIGO criteria. N/A = not applicable for dialysis group. Note: The combined lung-kidney transplant data (*n* = 2) can be mentioned in the text rather than the table to avoid complexity.

**Table 3:** Kidney transplant-specific characteristics (*n* = 18).

Variable	Kidney Transplant Recipients (*n* = 18)
Timing Variables	
Time from ESRD to kidney transplant (years), median (IQR)	0.5 (0.3–1.9)
Time from lung to kidney transplant (years), median (IQR)	6.8 (5.5–9.1)
Time from ESRD diagnosis to dialysis initiation (months), median (IQR)	0.35 (0.0–3.9)
Time on dialysis before kidney transplant (months), median (IQR) *	1.4 (0.2–5.0)
Donor and Surgical Characteristics	
Donor type, *n* (%)	
-Living donor	16 (88.9)
-Deceased donor	2 (11.1)
Cold ischemia time kidney (hours), mean ± SD	1.47 ± 1.39
Operative time kidney (minutes), mean ± SD	205 ± 34.8
Hospital stay kidney transplant (days), median (IQR)	8 (8–10)
Combined Transplants	
Combined lung-kidney transplant, *n* (%)	2 (11.1)
Total operative time for combined (minutes)	485 ± 78
ICU stay for combined cases (days)	7 (6–8)
Hospital stay for combined cases (days)	22 (18–26)

* For non-preemptive transplants only (*n* = 16).

**Table 4:** Long-term outcomes and follow-up.

Variable	Total (*n* = 32)	Dialysis (*n* = 14)	Kidney Transplant (*n* = 18)	*p*-Value
kidney Replacement Outcomes				
Dialysis dependency at 1 year, *n* (%) *	16 (66.7)	14 (100)	2 (11.1)	<0.001
Dialysis dependency at 3 years, *n* (%) **	15 (93.8)	14 (100)	1 (5.6)	<0.001
Kidney Graft Function (*n* = 18)				
Immediate graft function, *n* (%)	-	-	18 (100)	
Delayed graft function, *n* (%)	-	-	0 (0)	
Rejection episodes, *n* (%)	-	-	3 (16.7)	
eGFR at 1 year (mL/min/1.73 m^2^), mean ± SD ***	-	-	74.9 ± 26.2	
eGFR at 5 years (mL/min/1.73 m^2^), mean ± SD ****	-	-	57.0 ± 39.4	
Survival Outcomes				
Deaths during follow-up, *n* (%)	15 (46.9)	11 (78.6)	4 (22.2)	0.002
Median survival from lung transplant (months)	133.5	126.6	161.6	0.021
Cause of Death, *n* (%) ****				0.198
-Infection/Sepsis	8 (53.3)	7 (63.6)	1 (25.0)	
-Lung rejection	1 (6.7)	1 (9.1)	0 (0)	
-Other/Unknown	6 (40.0)	3 (27.3)	3 (75.0)	

* *n* = 24 patients evaluable at 1 year; ** *n* = 16 patients evaluable at 3 years; *** *n* = 17 patients with available data; **** *n* = 5 patients with available data; Total deaths *n* = 15. Renal replacement therapy outcomes, kidney graft function parameters, and mortality data stratified by treatment group. Denominators vary due to differential follow-up times and data availability, as noted.

**Table 5:** Univariate Cox regression analysis for mortality.

Variable	HR (95% CI)	*p*-Value
Kidney transplant (vs. dialysis) *	0.24 (0.08–0.75)	0.014
Age at lung transplant (per year) *	1.03 (1.00–1.06)	0.048
Male sex	0.68 (0.25–1.85)	0.449
Body Mass Index (per unit)	1.05 (0.91–1.21)	0.514
Smoking history	1.48 (0.53–4.13)	0.454
Time from lung to ESRD (per year)	0.98 (0.88–1.10)	0.754
AKI after lung transplant	1.19 (0.34–4.19)	0.786
Primary graft dysfunction grade ≥ 2	0.93 (0.26–3.28)	0.910
Early postoperative dialysis	0.64 (0.08–4.82)	0.663

Univariate Cox regression analysis for mortality hazard ratios for all-cause mortality from lung transplantation. * Variables with *p* < 0.10 were considered for multivariable analysis.

**Table 6:** Multivariate Cox regression analysis for mortality.

Variable	HR (95% CI)	*p*-Value
Kidney transplant (vs. dialysis) *	0.28 (0.09–0.89)	0.031
Age at lung transplant (per year) *	1.03 (1.00–1.06)	0.062

Multivariate Cox regression analysis for mortality: Age-adjusted hazard ratio for mortality comparing kidney transplantation versus dialysis. Model limited to two covariates based on sample size constraints (15 events). * Variables with *p* < 0.10 in univariate analysis were included in the multivariate model.

## Data Availability

The datasets used and/or analyzed during the current study are available from the corresponding author on reasonable request.

## Supplementary Materials

The following supporting information can be downloaded at: https://www.mdpi.com/article/10.3390/jcm14197017/s1. Figure S1: Overview of Lung Transplant Program Outcomes (1997–2025); Figure S2. Primary Pulmonary Diagnoses Leading to Lung Transplantation; Figure S3. Long-term Survival Following Lung Transplantation; Figure S4. Overview of Lung Transplant Program Outcomes (1997–2025); Table S1: Complete Statistical Analysis Summary; Table S2: Time Interval Analysis—Impact on Mortality; Table S3: Patient Flow and Timing; Table S4: Subgroup Analysis by Timing of Kidney Transplant; Table S5: Causes of Death by Treatment Group; Table S6: Model Diagnostics; Table S7: Sample Size Calculation for Future Studies. Tables S8–S10: These tables present exploratory, hypothesis-generating findings from a small single-center cohort that should not guide clinical practice but highlight patterns requiring validation in larger multicenter studies Table S8: Number Needed to Treat (NNT) Analysis; Table S9: Clinical Selection Score Components and Their Association with Treatment Allocation; Table S10: Clinical Phenotypes and Outcomes. References [19,20] are cited in the supplementary materials.

## Abbreviations

AKI:	Acute kidney injury
BMI:	Body mass index
CI:	Confidence interval
CKD:	Chronic kidney disease
CKD-EPI:	Chronic Kidney Disease Epidemiology Collaboration
CLAD:	Chronic lung allograft dysfunction
CMV:	Cytomegalovirus
CNI:	Calcineurin inhibitor
COPD:	Chronic obstructive pulmonary disease
eGFR:	Estimated glomerular filtration rate
EHR:	Electronic health record
ESRD:	End-stage renal disease
FEV1:	Forced expiratory volume in 1 s
HR:	Hazard ratio
ICD-10:	International Classification of Diseases, 10th Revision
ICU:	Intensive care unit
IPW:	Inverse probability weighting
IQR:	Interquartile range
IRB:	Institutional Review Board
ISHLT:	International Society for Heart and Lung Transplantation
KDIGO:	Kidney Disease: Improving Global Outcomes
NNT:	Number needed to treat
OPTN:	Organ Procurement and Transplantation Network
PSM:	Propensity score matching
RMST:	Restricted mean survival time
RRT:	Renal replacement therapy
SD:	Standard deviation
SMD:	Standardized mean difference
SRTR:	Scientific Registry of Transplant Recipients
