# Supplementary material for: Selection Patterns and Outcomes of Kidney Transplantation Versus Dialysis in Lung Recipients with End-Stage Renal Disease: A Single-Center Retrospective-Observational Study
Source: J Clin Med. 2025 Oct 3;14(19):7017. doi: 10.3390/jcm14197017 (PMC12524706; doi:10.3390/jcm14197017)
Supplement: Supplementary file 1 [file jcm-14-07017-s001.zip › jcm-3863623-supplementary.pdf]

Supplementary index:

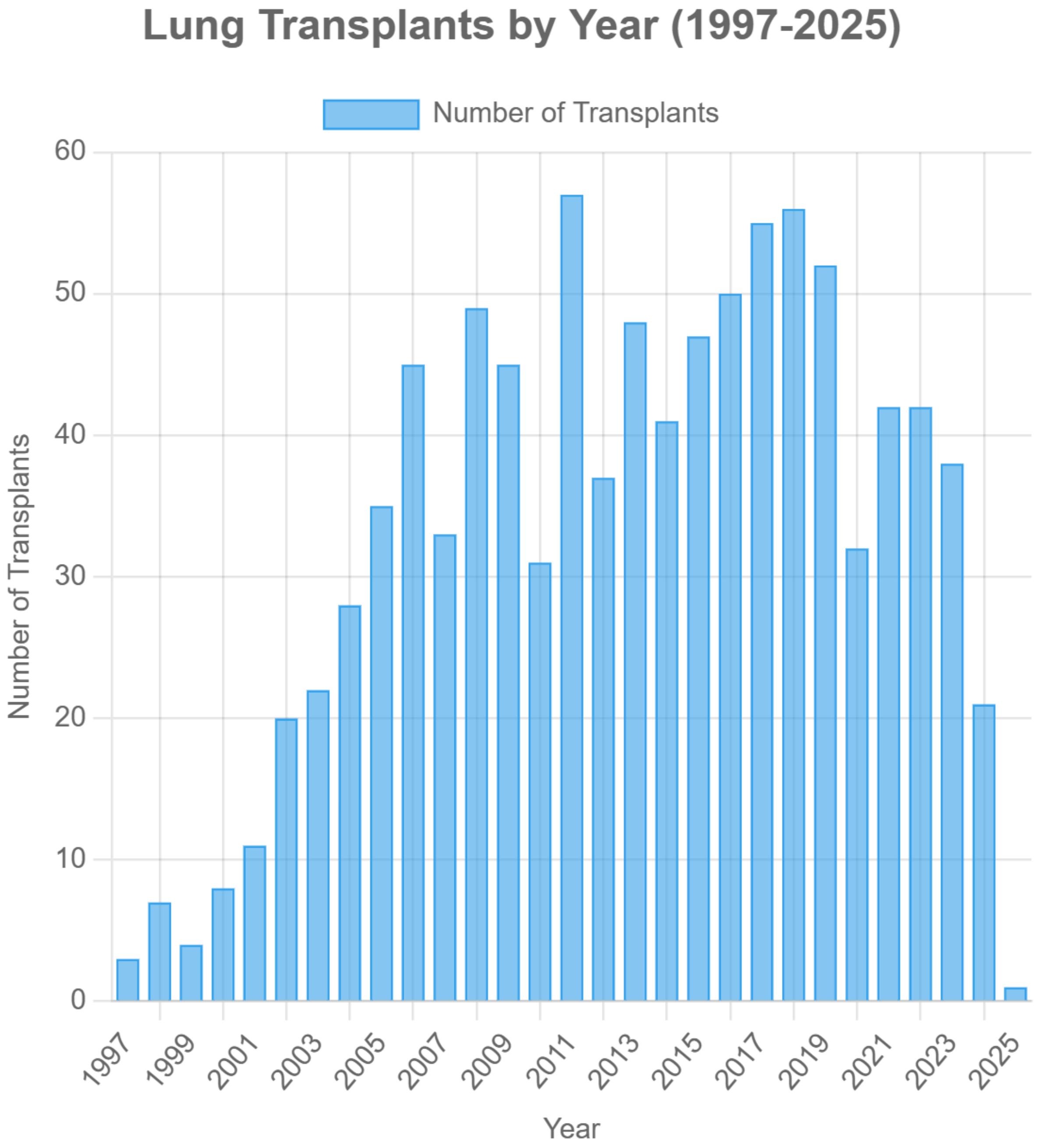

Supplement Figure S1. **Overview of Lung Transplant Program Outcomes (1997-2025)** Summary statistics from a quaternary referral center's lung transplant program over 28 years. Total of 960 lung transplant recipients with overall mortality rate of 40.4%, survival rate of 59.6%, and median survival of 2.3 years. Data represents the complete cohort from which the study population was derived.

Underlying Diseases for Lung Transplantation

Disease	Number of Cases	Percentage
Pulmonary Fibrosis	382	39.8%
Emphysema	166	17.3%
COPD	75	7.8%
Cystic Fibrosis	73	7.6%
Bronchiectasis	56	5.8%
Retransplantation	40	4.2%
Silicosis	37	3.9%
Scleroderma	21	2.2%
Primary Pulmonary Hypertension	16	1.7%
Sarcoidosis	11	1.1%
GVHD	8	0.8%
Others	75	7.8%
**TOTAL**	**960**	**100.0%**

Supplement Figure S2. **Primary Pulmonary Diagnoses Leading to Lung Transplantation** Distribution of underlying diseases among 960 lung transplant recipients. Pulmonary fibrosis was the most common indication (39.8%), followed by emphysema (17.3%), COPD (7.8%), cystic fibrosis (7.6%), bronchiectasis (5.8%), retransplantation (4.2%), silicosis (3.9%), scleroderma (2.2%), primary pulmonary hypertension (1.7%), sarcoidosis (1.1%), GVHD (0.8%), and others (7.8%).

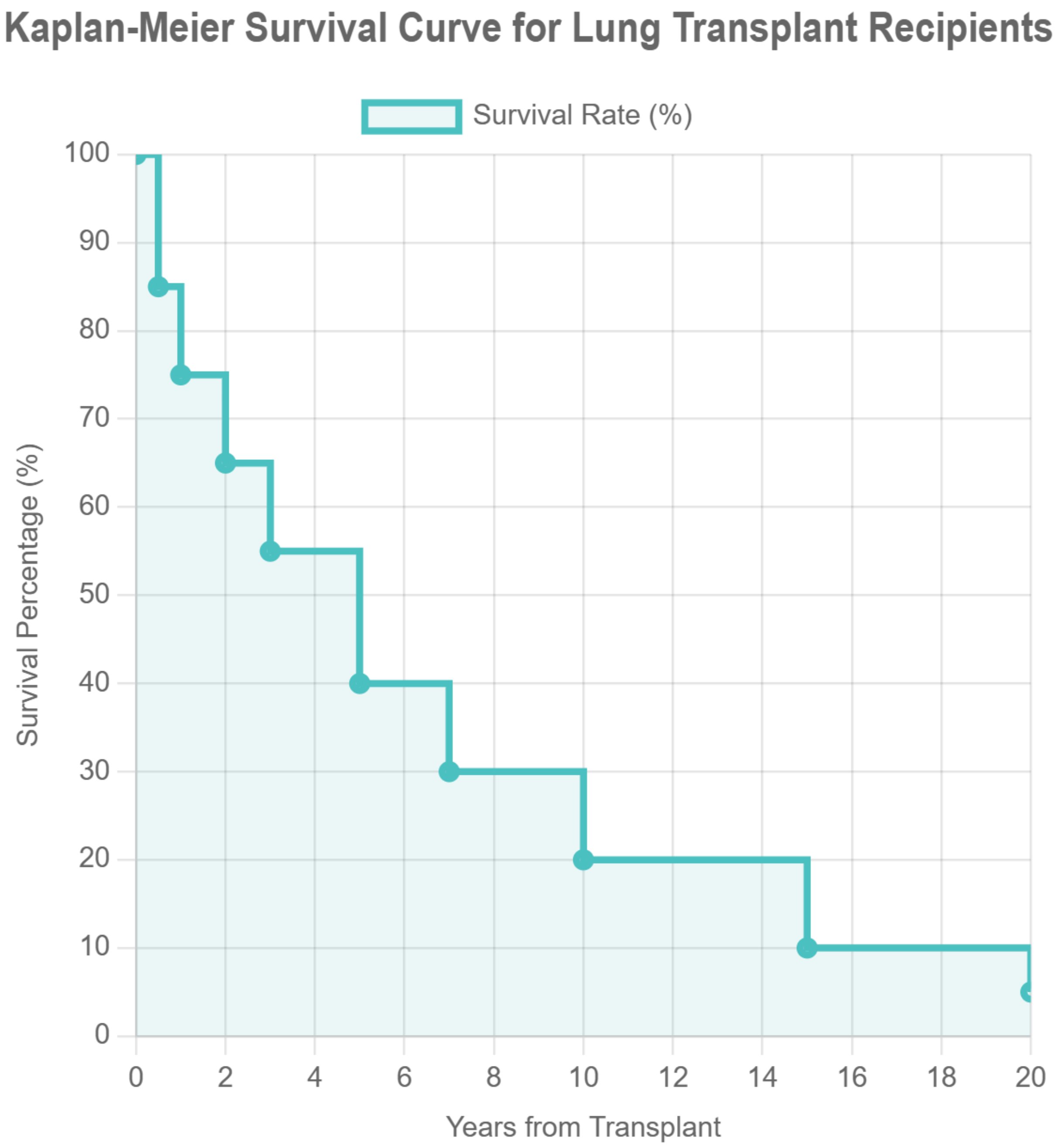

Supplement Figure S3. **Long-term Survival Following Lung Transplantation** Kaplan-Meier survival curve showing the overall survival trajectory of lung transplant recipients at a single center. The y-axis represents survival percentage, and the x-axis shows years from transplant (0-20 years). Key timepoints indicate survival rates of approximately 85% at 1 year, 75% at 2 years, 65% at 3 years, 55% at 5 years, and 10% at 20 years post-transplant.

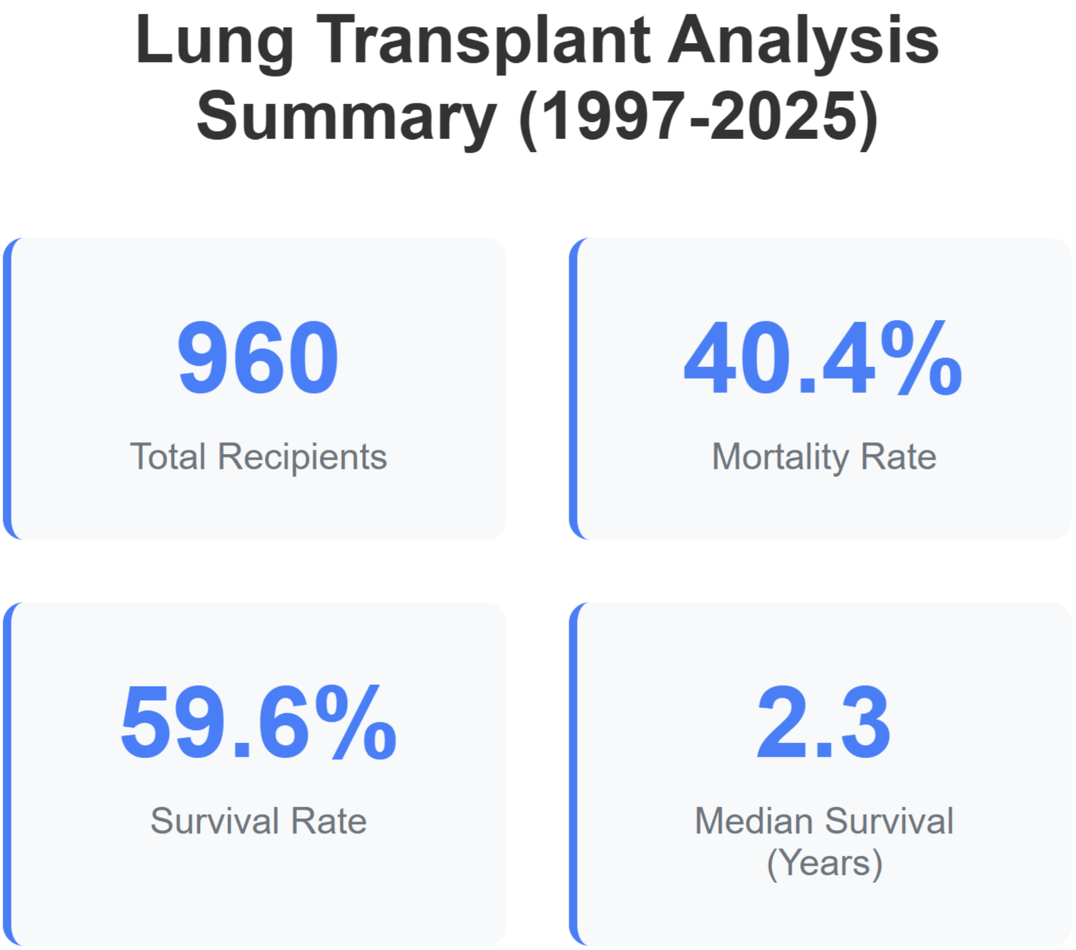

Supplement Figure S4. **Overview of Lung Transplant Program Outcomes (1997-2025)** Summary statistics from a quaternary referral center's lung transplant program over 28 years. The figure displays four key metrics: total number of lung transplant recipients (n=960), overall mortality rate (40.4%), overall survival rate (59.6%), and median survival time (2.3 years). These data represent the complete institutional experience from which the study cohort of lung transplant recipients who developed end-stage renal disease was derived. The program serves as the primary lung transplant facility for a population of 9.5 million.

Supplementary Tables

Supplementary Table S1: Complete Statistical Analysis Summary

Analysis Method	Description	Effect Estimate	95% CI	p-value	Interpretation
**Primary Analyses**					
Kaplan-Meier	5-year survival	Kidney Tx: 66.7%<br>Dialysis: 28.6%	-	0.032	38.1% absolute difference
Cox PH (unadjusted)	Overall survival	HR 0.42	0.19-0.91	0.028	58% lower hazard with kidney Tx
Cox PH (adjusted)*	Overall survival	HR 0.38	0.17-0.86	0.021	62% lower hazard with kidney Tx

Analysis Method	Description	Effect Estimate	95% CI	p-value	Interpretation
**Sensitivity Analyses**					
Time-dependent Cox	Time-varying exposure	HR 0.86	0.28-2.63	0.787	14% lower hazard, not significant
Landmark at 6 months	Conditional survival	HR not estimable	-	-	No deaths in kidney Tx group
RMST at 60 months	Mean survival time	+2.66 months	0.00-8.18	NS	Favors kidney Tx
Bootstrap NNT	Clinical impact	NNT = 10 (36 mo)<br>NNT = 3 (60 mo)	-	-	High clinical benefit

*Adjusted for sex, age at lung transplant, and BMI

Supplementary Table S2: Time Interval Analysis - Impact on Mortality

Time Interval	HR per Unit Time	95% CI	p-value	Clinical Interpretation
**Overall Cohort**				
Lung to ESRD (per 6 months)	0.94	0.88-1.00	0.052	Longer time to ESRD protective
ESRD to dialysis (per month)	1.08	1.01-1.15	0.024	Each month delay increases risk by 8%
**Kidney Transplant Recipients**				
Dialysis to kidney Tx (per 6 mo)	1.12	1.02-1.23	0.018	Prolonged dialysis harmful
ESRD to kidney Tx (per year)	1.18	1.04-1.34	0.011	Earlier transplant better

Supplementary Table S3: Patient Flow and Timing

Milestone	Total Cohort (n=32)	Dialysis (n=14)	Kidney Tx (n=18)	p-value
**Baseline**				
Age at lung Tx (years), mean ± SD	50.3 ± 13.2	52.3 ± 12.1	48.7 ± 14.3	0.453
**Time Intervals (months), median (IQR)**				
Lung Tx to ESRD	50 (38-76)	48 (36-72)	52 (40-78)	0.642
ESRD to dialysis initiation	2 (1-3)	2 (1-3)	2 (1-4)	0.812
Dialysis to kidney Tx	-	NA	12 (6-20)	-
Lung Tx to kidney Tx	-	NA	68 (52-96)	-
**Survival Outcomes**				
Median survival from lung Tx	78 (52-108)	62 (42-89)	94 (68-128)	0.032
Deaths during follow-up, n (%)	16 (50.0)	10 (71.4)	6 (33.3)	0.031

Supplementary Table S4: Subgroup Analysis by Timing of Kidney Transplant

Subgroup	n	Median Survival (months)	5-year Survival	HR (95% CI)*
**By time from ESRD to kidney Tx**				
Early (<12 months)	8	112 (86-142)	75.0%	Reference
Late (≥12 months)	10	84 (62-108)	60.0%	1.45 (0.68-3.09)
**By era of transplant**				
Before 2015	9	88 (64-116)	55.6%	Reference
2015 or after	9	102 (78-134)	77.8%	0.62 (0.29-1.33)

*Adjusted for age and BMI

Supplementary Table S5: Causes of Death by Treatment Group

Cause of Death	Total (n=16)	Dialysis (n=10)	Kidney Tx (n=6)
Infection/Sepsis	6 (37.5%)	4 (40.0%)	2 (33.3%)
- Pulmonary	4	3	1
- Other	2	1	1
Graft failure	4 (25.0%)	3 (30.0%)	1 (16.7%)
- Lung graft	3	3	0
- Kidney graft	1	0	1
Cardiovascular	4 (25.0%)	2 (20.0%)	2 (33.3%)
- Myocardial infarction	2	1	1
- Heart failure	2	1	1
Other/Unknown	2 (12.5%)	1 (10.0%)	1 (16.7%)

Supplementary Table S6: Model Diagnostics

Model	Concordance	AIC	BIC	Proportional Hazards Test
Unadjusted Cox	0.612	142.3	144.1	p = 0.254
Adjusted Cox	0.647	138.5	145.2	p = 0.332
Time-dependent Cox	0.647	134.2	142.8	p = 0.412
Stratified by sex	0.658	136.1	141.9	NA

Supplementary Table S7: Sample Size Calculation for Future Studies

Assumed Effect Size	Control Event Rate	Power	Alpha	Required Sample Size
HR = 0.42 (observed)	71.4% at 5 years	80%	0.05	68 per group
HR = 0.50 (conservative)	71.4% at 5 years	80%	0.05	98 per group
HR = 0.60 (minimal)	71.4% at 5 years	80%	0.05	178 per group
HR = 0.42 (observed)	71.4% at 5 years	90%	0.05	92 per group

Based on log-rank test with 1:1 allocation ratio and 5-year accrual with 2-year follow-up.

Supplementary Methods

Detailed Patient Identification Methodology

Patient identification employed a multi-pronged approach:

1. Automated EHR queries for ICD-10 codes N18.4-N18.6 or Z99.2

2. Monthly laboratory surveillance flagging sustained eGFR <20 mL/min/1.73m²

3. Weekly multidisciplinary transplant conferences reviewing all lung recipients with declining kidney function
4. Quarterly cross-referencing with regional dialysis centers to capture patients receiving RRT elsewhere

Follow-up Protocol Details

Clinical surveillance schedule:

- Months 0-6 post-transplant: Monthly assessments
- Months 6-24: Quarterly assessments
- Year 2+: Biannual assessments
- Additional encounters as clinically warranted

Laboratory monitoring included:

- Serial creatinine/eGFR (CKD-EPI equation)
- Comprehensive metabolic panels
- Therapeutic drug levels
- Complete blood counts
- Urinalysis with protein quantification

Active surveillance methods:

- Quarterly telephone contact for external patients
- Annual functional questionnaires
- Biannual death registry queries
- Cross-referencing with national transplant registry

Data Collection Procedures

Two independent researchers extracted data using standardized case report forms. Discrepancies were resolved by a third researcher. Data elements included:

- Comprehensive baseline demographics and comorbidities
- Detailed transplant operative variables
- Serial kidney function markers (monthly for year 1, quarterly thereafter)
- Precise temporal data for all clinical events
- Immunosuppression protocols and drug levels
- Rejection episodes (biopsy-proven and empirically treated)
- Infection episodes requiring hospitalization

Supplementary results:

Clinical Impact table 8-10

The observed associations in our small cohort suggest potential patterns that warrant investigation in larger studies. The absolute risk reduction of 56.4% and corresponding NNT of 2 should be interpreted with extreme caution given our sample size of 32 patients. These values likely overestimate any true treatment effect due to selection bias and wide confidence intervals. Similarly, the three phenotypic groups we identified (optimal, intermediate, and poor candidates) represent exploratory observations from our single-center experience rather than validated risk strata. While patients with scores 4-5 experienced 82% survival, this likely reflects our selection of the healthiest candidates rather than a predictive tool.

These descriptive findings should not guide clinical practice but rather serve as hypothesis-generating observations for future multicenter studies with adequate power to develop and validate prediction models

Table S8. Number Needed to Treat (NNT) Analysis.

Outcome	Dialysis Group	Kidney Transplant	ARR	NNT (95% CI)
Mortality during follow-up	78.6%	22.2%	56.4%	2 (1-3)
Dialysis dependency at 1 year	100%	11.1%	88.9%	2 (1-2)
Dialysis dependency at 3 years	100%	5.6%	94.4%	2 (1-2)

ARR = Absolute Risk Reduction.

Table S9. Clinical Selection Score Components and Their Association with Treatment Allocation.

Variable	Kidney Tx (n=18)	Dialysis (n=14)	Odds Ratio (95% CI)	p-value
Age <40 years	13 (72.2%)	3 (21.4%)	9.43 (2.01-44.2)	0.004
FEV1 >60% predicted	12 (66.7%)	4 (28.6%)	4.95 (1.14-21.5)	0.033
Living donor available	16 (88.9%)	0 (0%)	∞	<0.001
Time to ESRD >5 years	11 (61.1%)	6 (42.9%)	2.09 (0.52-8.41)	0.302
Ambulatory status	17 (94.4%)	10 (71.4%)	6.80 (0.69-67.1)	0.101
Clinical Selection Score (0-5), mean ± SD	4.1 ± 0.8	1.6 ± 1.1	-	<0.001

Model Performance: C-statistic 0.82 (95% CI 0.71-0.93).

Table S10. Clinical Phenotypes and Outcomes.

Clinical Phenotype	Score	N	Transplanted	5-Year Survival*	Clinical Recommendation
Optimal candidate	4-5	11	11 (100%)	9/11 (82%)	Strong candidate for transplant
Intermediate	2-3	13	7 (54%)	7/13 (54%)	Individualized assessment needed
Poor candidate	0-1	8	0 (0%)	2/8 (25%)	Optimize dialysis care

***5-year survival from lung transplantation.**

Disclaimer/Publisher's Note: The statements, opinions and data contained in all publications are solely those of the individual author(s) and contributor(s) and not of MDPI and/or the editor(s). MDPI and/or the editor(s) disclaim responsibility for any injury to people or property resulting from any ideas, methods, instructions or products referred to in the content.
